# Glutathione S-transferase A2 promotes hepatocellular carcinoma recurrence after liver transplantation through modulating reactive oxygen species metabolism

**DOI:** 10.1038/s41420-021-00569-y

**Published:** 2021-07-21

**Authors:** Kevin Tak-Pan Ng, Oscar Wai-Ho Yeung, Yin Fan Lam, Jiang Liu, Hui Liu, Li Pang, Xin Xiang Yang, Jiye Zhu, Weiyi Zhang, Matthew Y. H. Lau, Wen Qi Qiu, Hoi Chung Shiu, Man Kit Lai, Chung Mau Lo, Kwan Man

**Affiliations:** Department of Surgery, HKU-SZH & LKS Faculty of Medicine, The University of Hong Kong, Hong Kong SAR, China

**Keywords:** Prognostic markers, Hepatocellular carcinoma

## Abstract

Hepatocellular carcinoma (HCC) recurrence after liver transplantation remains a significant clinical problem. Ischemia-reperfusion injury (IRI) occurred inevitably at the early phase after liver transplantation (LT) spawns a significant risk of HCC recurrence. However, their linkage and IRI-derived risk factors for HCC recurrence remain exclusive. Understanding the mechanism of post-transplantation hepatic injury could provide new strategies to prevent the later event of HCC recurrence. We demonstrated that glutathione S-transferase A2 (GSTA2) expression was significantly associated with early phase hepatic and systemic injury and ROS level after liver transplantation. Early phase circulating GSTA2 (_EPC_GSTA2) protein was a significant predictor of HCC recurrence and survival. Heterogeneous single nucleotide polymorphism at G335C of GSTA2 was significantly associated with poor survival of HCC recipients. Enhancement of GSTA2 could protect HCC cells against H_2_O_2_-induced cell death by compensating for the elevated ROS stress. We also demonstrated that GSTA2 played crucial roles in regulating the ROS-associated JNK and AKT signaling pathways and ROS metabolism in HCCs in responding to a dynamic ROS environment. Functionally, endogenous or exogenous upregulation of GSTA2 could promote HCC growth and invasion through activating the epithelial–mesenchymal-transition process. Targeted inhibition of GSTA2 could suppress HCC growth and metastasis. In conclusion, GSTA2 could be a novel prognostic and therapeutic target to combat HCC recurrence after liver transplantation.

## Introduction

Liver transplantation (LT) is the best curative treatment for hepatocellular carcinoma (HCC) patients under selection criteria [1]. However, HCC recurrence is the main reason leading to the poor overall survival of HCC recipients after LT due to the lack of effective prediction and prevention strategies for HCC recurrence [2]. Understanding the mechanisms of this recurred disease is a pressing need to provide clues for identifying novel predictive molecules and developing novel preventive strategies. Ischemia–reperfusion injury (IRI) is an unavoidable consequence after LT, especially at the early phase. Clinical and experimental studies have indicated that IRI potentially increases the risk of HCC recurrence after LT [3–5]. Our previous study has also demonstrated that increased hepatic IRI exaggerates liver tumor progression and increases the likelihood of lung metastasis through activating cell adhesion, invasion, and angiogenesis pathways [6]. Our studies have also indicated that an elevated early phase hepatic injury after LT can alter the microenvironment and signaling pathways that not only favors tumor development but also promotes the invasiveness of tumor cells [7–9]. There are still many opening questions needed to be clarified including the molecular mechanisms linking early phase IRI and late-phase HCC recurrence after LT, and which IRI-derived factors are a critical risk for triggering HCC recurrence.

Hepatic IRI is closely linking to the production of reactive oxygen species (ROS) whose augmentation is one of the major initiators in triggering hepatic damage especially in the early stage of IRI [10]. Aggravated hepatic IRI further elevates the production of ROS from inflammatory cells and mitochondria [11]. ROS can trigger the activation of multiple cellular signal pathways which in turn regulate the level of intracellular ROS in order to maintain the homeostasis of ROS for proper physiological functions [12]. The c-Jun N-terminal kinases (JNK) pathway which is a critical ROS-targeting signaling pathway regulates the activity of multiple transcriptional factors upon activation. A higher level of ROS can activate the JNK pathway and prolong the activation of the JNK pathway [12]. On the other hand, ROS can directly activate PI3K signaling cascade and inactivate phosphatase and tensin homolog (PTEN) followed by inhibiting the activation of AKT [13]. Generally, cells are composed of three important pathways to eliminate ROS toxicity including reduced glutathione, thioredoxin, and catalase [14, 15]. Glutathione S-transferase A2 (GSTA2) which is a phase II detoxifying enzyme is highly expressed in the liver tissue functioning in detoxification of electrophilic compounds, such as products of oxidative stress [16]. GSTA2 can regulate glutathione utilization to protect cells against ROS damage [16]. Overexpression of GSTA2 protects cancer cells against apoptosis from chemotherapeutic drugs [17]. The polymorphic feature of GSTA2 is associated with different diseases and cancers [18–20]. Upregulation of GSTA2 is associated with the doxorubicin resistance of HCC [21]. In this study, GSTA2 was identified to be the most significantly upregulated gene of HCC recipients who developed recurred HCC after LT. We aimed to characterize the clinical significance and prognostic value of GSTA2 in HCC recipients and to investigate its functions and underlying mechanisms in HCC recurrence and metastasis.

## Results

### Identification of GSTA2 as an HCC-recurrence associated gene at early phase after LT

**Fig. 1:**
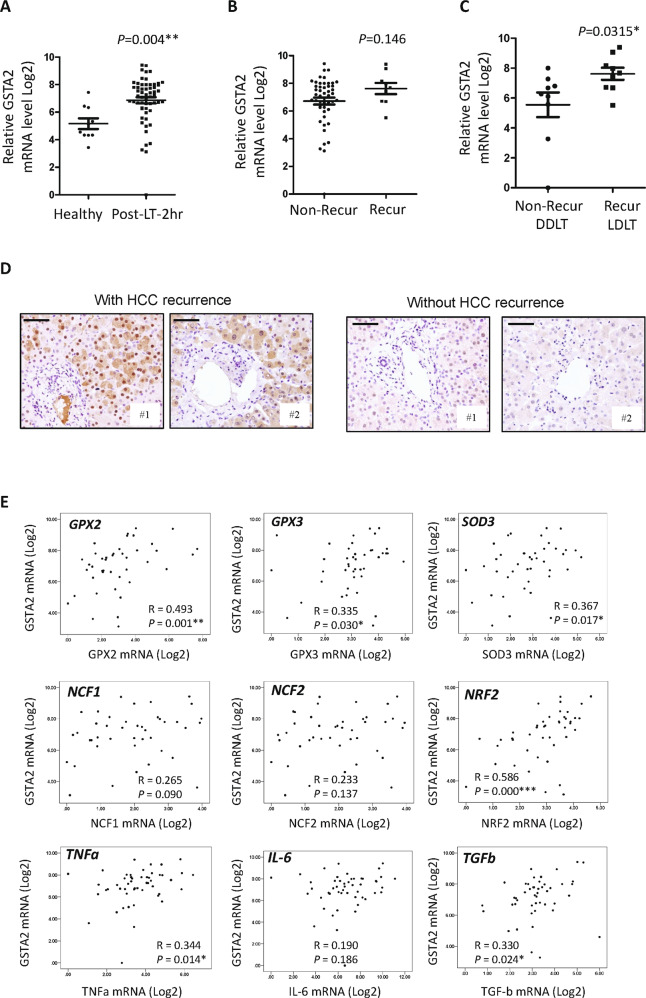
Expression analysis of hepatic GSTA2 at 2-hr after liver transplantation. **A** Expression level of hepatic GSTA2 mRNA in the healthy donors and in the recipients at 2-hour after liver transplantation. **B** Comparison of post-LT 2-hr hepatic GSTA2 mRNA between recipients with and without post-LT HCC recurrence. **C** The expression level of hepatic GSTA2 mRNA in LDLT recurrent recipients and in DDLT non-recurrent recipients. **D** IHC analysis of GSTA2 protein between post-LT recurrent and non-recurrent recipients. Scale bar: 100 μM. Recur, post-LT HCC recurrence; non-recur, without post-LT HCC recurrence. **E** Correlation analysis of post-LT hepatic GSTA2 mRNA and ROS-associated genes (GPX2, GPX3, SOD3, NCF1, NCF2, and NRF2) and injury-associated genes (TNFa, IL-6, and TGFb). *P < 0.05; **P < 0.01.

RNA sequencing analysis revealed that GSTA2 was the most upregulated gene in the HCC recipients with HCC recurrence compared to non-HCC recurrent recipients (Supplementary Table S2). The expression level of hepatic GSTA2 mRNA at the post-LT early phase was significantly higher than in the healthy donors (Fig. 1a). The expression level of hepatic GSTA2 mRNA in recurrent recipients was higher than in non-recurrent recipients (Fig. 1b). A significantly higher expression of GSTA2 mRNA was determined in LDLT recurrent recipients compared to DDLT non-recurrent recipients (Fig. 1c). GSTA2 protein in the liver of recurrent recipients after LDLT was higher than in non-recurrent recipients after DDLT (Fig. 1d). Upregulation of hepatic GSTA2 mRNA at early phase was significantly correlated with genes related to ROS metabolism (GPX2, GPX3, SOD3, and NRF2 mRNAs) and hepatic injury (TNFα and TGFβ mRNAs) (Fig. 1e).

### Early phase circulating GSTA2 (_EPC_GSTA2) protein was an indicator of hepatic injury and a prognostic biomarker of HCC recurrence

**Fig. 2:**
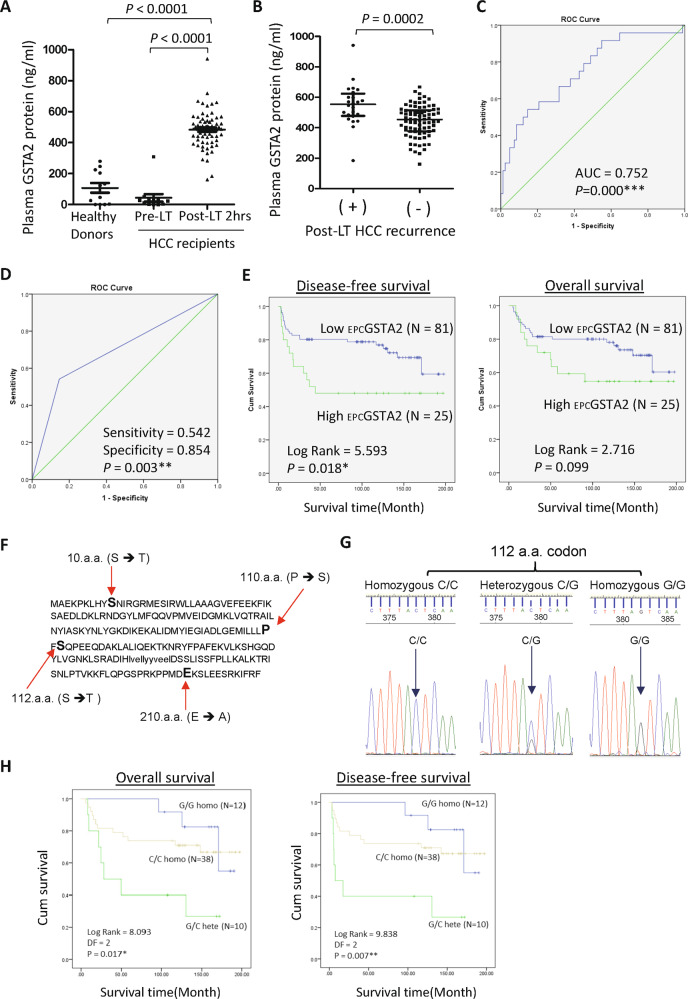
Post-liver transplantation early phase GSTA2 associated with late-phase HCC recurrence. **A** The concentration of plasma GSTA2 protein in healthy donors and in pretransplantation (Pre-LT) and in post-transplantation early phase (Pre-LT 2 hr) of HCC recipients. **B** The level of early phase plasma GSTA2 protein in Recurrent recipients was significantly higher than in the non-recurrence group. **C** ROC analysis of post-transplant early phase plasma GSTA2 protein in predicting post-transplant HCC recurrence. **D** ROC analysis of High plasma GSTA2 protein in predicting HCC recurrence after liver transplantation. **E** Kaplan–Meier analysis of _EPC_GSTA2 in disease-free survival and overall survival of HCC recipients after liver transplantation. **F** Polymorphic amino acid residues in the GSTA2 protein of the transplanted liver of HCC recipients. **G** Sanger sequencing chromatographs of C335G homozygous and heterozygous alleles in GSTA2 coding sequencing. **H** Kaplan–Meier analysis of G335C SNP homozygosity and homozygosity of GSTA2 coding sequencing in the transplanted liver in predicting overall and disease-free survival of HCC recipients after liver transplantation. *P < 0.05; **P < 0.01.

A significantly higher level of _EPC_GSTA2 protein was detected in HCC recipients (*N* = 106) compared to the healthy donors and in pretransplant plasma of the recipients (Fig. 2a). The _EPC_GSTA2 protein was not significantly correlated with the pretransplantation clinicopathological characteristics of the recipients, but significantly correlated with the duration of warm ischemic time, the levels of AST and ALT, ROS level, IL-10 and IL-8 proteins in early phase plasma of HCC recipients (Table 1). Recipients with post-LT HCC recurrence were detected with a significantly higher concentration of _EPC_GSTA2 protein compared to recipients without developing HCC recurrence (Fig. 2b). The _EPC_GSTA2 protein was significantly correlated with early phase plasma miR-1246 which is associated with post-transplantation HCC recurrence [31]. The _EPC_GSTA2 protein could significantly discriminate post-LT HCC recurrence with the area under the curve value of 0.752 (*P* = 0.000, Fig. 2c). High _EPC_GSTA2 protein was a significant predictor of post-LT HCC recurrence (*P* = 0.003; Fig. 2d). Among those significant factors, _EPC_GSTA2 protein and UCSF criteria were independent predictors in predicting HCC recurrence (Table 2). Moreover, high _EPC_GSTA2 protein was significantly associated with poor disease-free survival of HCC patients after LT (Fig. 2e, Table 2).

**Table 1:** Statistical analysis of early phase GSTA2 protein with clinical and experimental variables of HCC recipients.

Variables	Early phase GSTA2	*P* value
*Pretransplantation*		
Sex		
Male (*N* = 88)	486.6 (183.9–718.4)	0.160
Female (*N* = 18)	436.7 (160.9–941.1)	
Age		
≤57	484.3 (230.0 – 941.1)	0.493
>57	478.5 (160.9 – 667.8)	
HbsAg before LT		
Positive (*N* = 85)	486.3 (160.9–941.1)	0.252
Negative (*N* = 21)	455.6 (230.0–667.8)	
Milan criteria		
Within (*N* = 59)	477.8 (160.9–718.4)	0.233
Beyond (*N* = 47)	496.9 (183.9–941.1)	
UCSF criteria		
Within (*N* = 74)	470.2 (160.9–718.4)	0.173
Beyond (*N* = 32)	499.1 (183.9–941.1)	
AFP level before LT		
≤400 ng/ml (*N* = 89)	477.0 (160.9–941.1)	0.615
>400 ng/ml (*N* = 17)	496.9 (242.0–632.2)	
Type of graft		
Live graft (*N* = 81)	477.0 (160.9–718.4)	0.529
Cadaveric graft (*N* = 25)	501.4 (256.5–941.1)	
Graft weight to recipient ESLV (%)	*R* = 0.113	0.248
Pre–LT AST (μ/l, *N* = 106)	*R* = 0.127	0.195
Pre-LT ALT (μ/l, *N* = 106)	*R* = 0.067	0.495
*During and post-transplantation*		
Cold ischemic time (min) (*N* = 106)	*R* = 0.182	0.062
Warm ischemic time (min (*N* = 104)	*R* = 0.218	0.026*
Post-LT ROS level (*N* = 69)	*R* = *0.578*	0.000***
Post –LT AST (μ/l, *N* = 106)	*R* = 0.268	0.022*
Post-LT ALT (μ/l, *N* = 106)	*R* = 0.300	0.031*
Post-LT bilirubin (mg/dl, *N* = 72)	*R* = 0.037	0.758
Post-LT-2 h GM-CSF (*N* = 100)	*R* = -0.108	0.283
Post-LT-2 h IFN-alpha2 (*N* = 100)	*R* = 0.060	0.550
Post-LT-2 h IFN-gamma (*N* = 100)	*R* = 0.030	0.768
Post-LT-2 h IL-10 (*N* = 100)	*R* = 0.359	0.000***
Post-LT-2 h IL-6 (*N* = 100)	*R* = 0.052	0.606
Post-LT-2 h IL-8 (*N* = 100)	*R* = 0.306	0.002**
Post-LT-2 h IP-10 (*N* = 100)	*R* = 0.120	0.234
Post-LT-2 h MCP-1 (*N* = 100)	*R* = 0.105	0.297
Post-LT-2 h TNF-alpha (*N* = 100)	*R* = 0.026	0.795

*P < 0.05.

**P < 0.01.

***P < 0.001.

**Table 2:** Prognostic value of early phase circulating GSTA2 protein in HCC recipients after liver transplantation.

1. Prediction of HCC recurrence						
	ROC analysis				Logistics Regression	
Factors	Sensitivity (%)	Specificity (%)	AUC (95% CI)	*P* value	HR	*P* value
Early phase plasma GSTA2 protein (high vs. low)	45.8	85.4	0.70 (0.57–0.83)	0.003**	9.034	0.002**
Milan Criteria (beyond vs. within)	83.3	67.1	0.75 (0.65–0.86)	0.000***	1.046	0.966
UCSF criteria (beyond vs. within)	75.0	82.9	0.79 (0.68–0.90)	0.000***	7.532	0.035*
Tumor size (≥3 cm vs. <3 cm)	82.6	52.4	0.68 (0.56–0.79)	0.010**	3.148	0.141
Vascular permeation (yes vs. no)	59.1	79.3	0.69 (0.56–0.82)	0.006**	3.773	0.062
Pre-OT AFP level (≥400 ng/ml vs. <400 ng/ml)	33.3	88.9	0.61 (0.47–0.75)	0.097	N/A	
Graft weight to recipient ESLV (<60% vs. >60%)	75.0	37.8	0.56 (0.44–0.69)	0.342	N/A	
Type of transplant (LDLT vs. DDLT)	79.2	24.4	0.52 (0.39–0.65)	0.792	N/A	
Tumor number (>3 vs. <3)	34.8	89.0	0.62 (0.48–0.76)	0.082	N/A	
Differentiation (poor vs. well)	23.8	94.9	0.59 (0.45–0.74)	0.190	N/A	

2. Prediction of disease-free survival				
	Disease-free survival			
Factors	Univariate regression		Multivariate regression	
	HR (95% CI)	*P* value	HR (95% CI)	*P* value
Plasma GSTA2 protein (high vs. low)	2.24 (1.13–4.45)	0.021*	1.75 (0.81–3.75)	0.154
Sex (male vs. female)	1.04 (0.43–2.51)	0.928		
Age (≤55 yr vs. <55 yr)	1.06 (0.54–2.08)	0.860		
Serum AFP (>400 ng/ml vs. ≤400 ng/ml)	2.89 (1.37–6.07)	0.005**	1.73 (0.70–4.26)	0.234
Tumor size (>3 cm vs. ≤3 cm)	1.96 (0.95–4.02)	0.068		
Tumor number (>3 vs. ≤3)	2.00 (0.09–4.44)	0.088		
Vascular permeation (yes vs. no)	2.36 (1.19–4.68)	0.014*	1.246 (0.54–2.87)	0.606
Differentiation (poor vs. well)	3.08 (1.26–7.54)	0.013*	1.90 (0.73–4.95)	0.191
UCSF criteria (beyond vs. within)	4.22 (2.13–8.37)	0.000***	2.30 (1.00–5.31)	0.050
Milan Criteria (beyond vs. within)	3.09 (1.53–5.25)	0.002**	N/A	
Type of LT (DDLT vs. LDLT)	1.30 (0.61–2.78)	0.500	N/A	
Graft size (>60% vs. ≤60%)	0.75 (0.38–1.47)	0.402	N/A	

^*^P < 0.05.

^**^P < 0.01.

^***^P < 0.001.

### GSTA2 S112T polymorphism associated with HCC recurrence and survival

There were 12 differential SNPs in GSTA2 transcript identified by RNA sequencing between recipients with and without HCC recurrence (Supplementary Table S3). There were five SNPs within the coding sequence of the GSTA2 transcript. In which four SNPs were non-synonymous. Sanger sequencing analysis of full length of GSTA2 cDNA (encoding 222 amino acids) was conducted on the liver biopsies collected from the early phase of 60 HCC recipients. Four SNPs were detected at the positions of 28(T > A), 329(C > T), 335(G > C), and 629(A > C), which were either heterozygous or homozygous non-synonymous SNPs (Fig. 2f). Among all the detected SNPs, the G335C SNP (Fig. 2g) which results in residue S112T substitution was found to be significantly different between HCC recipients with and without HCC recurrence (*P* = 0.002, Supplementary Table S4). The percentage of heterogeneous SNP G335C in the recipients with HCC recurrence was nearly fivefold higher than the recipients without HCC recurrence. Importantly, G335C heterogeneous G/C alleles of GSTA2 transcript were significantly associated with poor overall (*P* = 0.017) and disease-free survival of HCC recipients (*P* = 0.007, Fig. 2h).

### The protective role of GSTA2 for HCCs against high oxidative stress-induced cell death

**Fig. 3:**
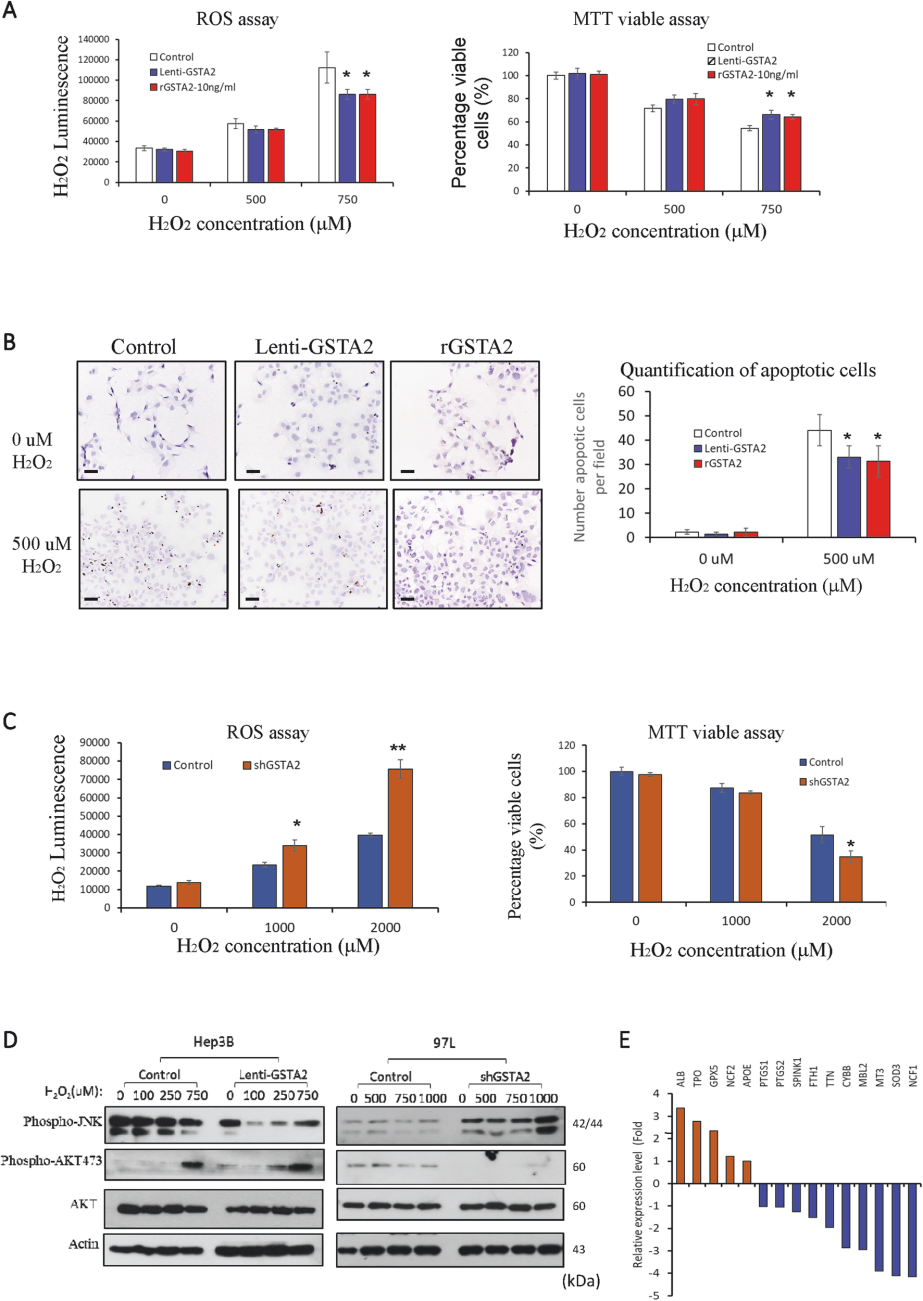
The mechanism of GSTA2 in regulating ROS embolism of HCC. **A** ROS assay and MTT assay of Hep3B cells with upregulated expression of GSTA2 (Lenti-GSTA2) or administration of human recombinant GSTA2 protein (rGSTA2) under different concentrations of H_2_O_2_. **B** TUNEL apoptosis assay of Hep3B cells with upregulated expression of GSTA2 (Lenti-GSTA2) or administration of human recombinant GSTA2 protein (rGSTA2) under H_2_O_2_ condition. Scale bar: 100 μM. **C** ROS assay and MTT assay for 97L-Control and 97L-shGSTA2 cells under different concentrations of H_2_O_2_. **D** Western blot analysis of JNK and AKT pathway in GSTA2-overexpressing and GSTA2-suppressing HCC cells under H_2_O_2_ conditions. **E** Differentially expressed ROS-associated genes by RT^2^ PCR array analysis in MHCC97L-shGSTA2 cells and MHCC97L-control cells under H_2_O_2_ condition. *P < 0.05; **P < 0.01.

The normal hepatocyte cell line could elevate intracellular ROS in responding to the increased concentrations of H_2_O_2_ (Supplementary Fig. S1A). High ROS levels caused a harmful effect on normal hepatocytes, leading to a reduction of proliferation rate (Supplementary Fig. S1B). The administration of human recombinant GSTA2 protein could help the normal hepatocytes to compensate for the H_2_O_2_-induced upregulation of ROS and reduce the anti-proliferative effect under a high ROS condition (Supplementary Fig. S1). Meanwhile, administration of recombinant GSTA2 protein or forced overexpression of GSTA2 gene in HCC cells could contribute a compensation effect on H_2_O_2_-induced ROS elevation (ROS assay in Fig. 3a). HCC cells with either an increased level of exogenous or endogenous GSTA2 exhibited a significantly higher proliferation rate than the control cells under a high-ROS condition (MTT assay in Fig. 3a). Apoptosis assay revealed that the upregulation of GSTA2 expression, either by exogenous or endogenous means, could protect the HCC cells against high H_2_O_2_-induced apoptosis (Fig. 3b). In addition, suppression of GSTA2 expression in the metastatic HCC cells further intensified the effect of H_2_O_2_, leading to a significantly higher intracellular ROS level and lower proliferation rate (Fig. 3c).

### GSTA2 regulated ROS metabolism of HCC

Two important ROS-mediating signaling pathways were characterized in GSTA2-overexpressing and GSTA2-suppressing HCC cells. Overexpression of GSTA2 in HCC cells suppressed its ROS-mediated activation of the JNK pathway, while suppression of GSTA2 enhanced the activation of the JNK pathway of HCC cells under an increased ROS condition (Fig. 3d). Overexpression of GSTA2 in HCC cells enhanced the activation of the AKT pathway under ROS-stimulation, while suppression of GSTA2 inhibited the activation of the AKT pathway in HCC cells (Fig. 3d). The effect of GSTA2 on the expression of 84 ROS-associated genes under the H_2_O_2_ environment was studied by RT^2^ PCR array analysis. By comparing their expressions between MHCC97L-shGSTA2 and MHCC97L-control cells, there were five upregulated and ten downregulated genes related to oxidative stress were identified in MHCC97L-shGSTA2 cells under H_2_O_2_ condition (Fig. 3e). Among them, eight of them are involved in ROS metabolism including TPO, SOD3, NCF1, NCF2, APOE, FTH1, GPX5, and MBL2. The correlation analysis of the expression of GSTA2 and the identified ROS-associated genes using the LIHC database in the GEPIA web server revealed that the expression level of GSTA2 was significantly correlated with ALB, TPO, APOE, MBL2, and FTH1 in the tumor tissues (Supplementary Fig. S2).

### GSTA2 positively associated with the aggressiveness of HCC

**Fig. 4:**
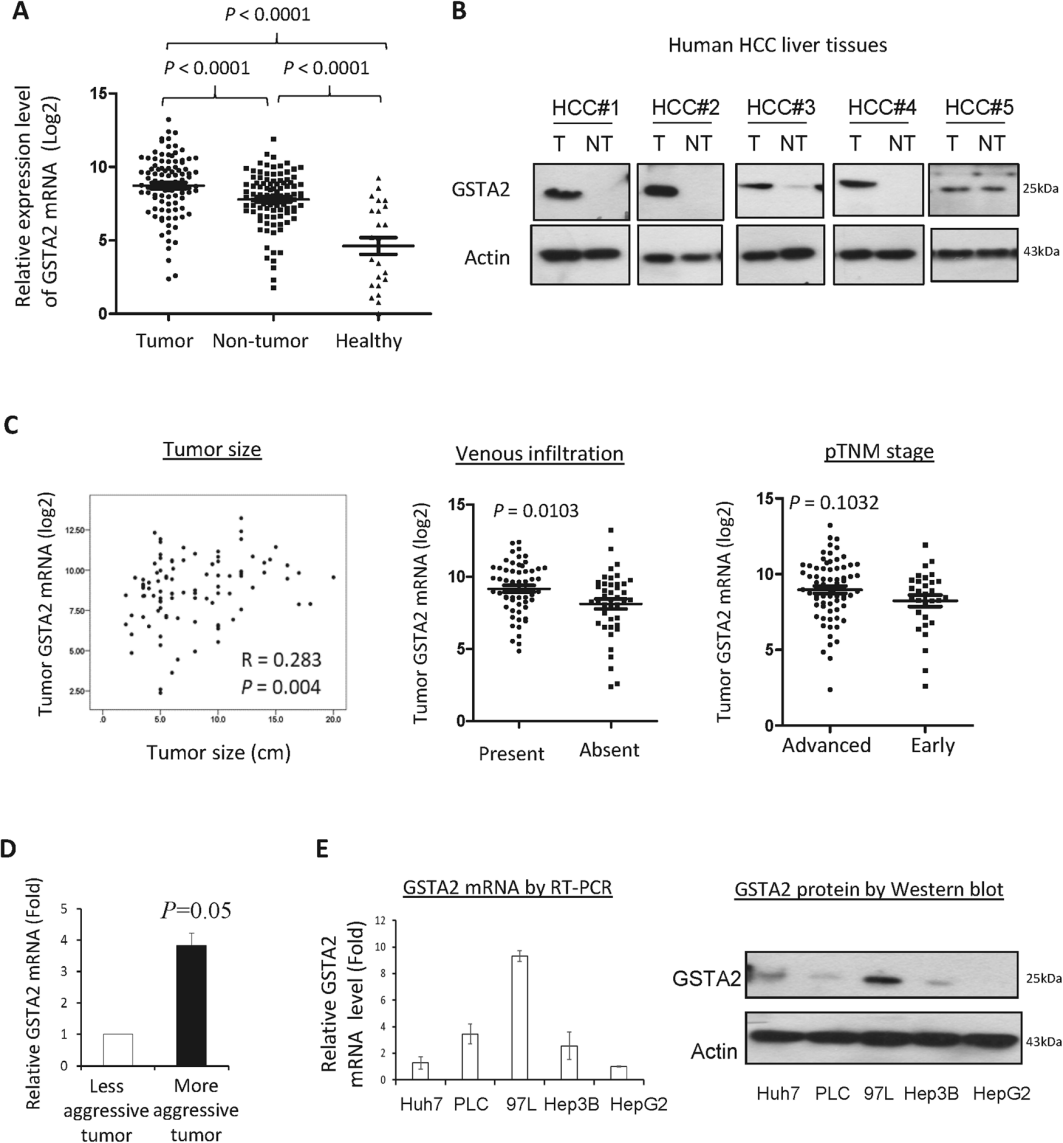
The expression significance of GSTA2 in HCC. **A** Expression of GSTA2 mRNA in the tumor (T) and non-tumor (NT) liver tissues of HCC patients, and in the liver tissues of healthy donors. **B** Western blot analysis of GSTA2 protein in paired tumor (T) and non-tumor (NT) tissues of HCC patients. **C** The correlation analysis of the expression level of tumoral GSTAs mRNA and tumor size, the presence of venous infiltration, and pTNM stage of HCC patients. **D** The expression level of tumoral GSTA2 mRNAs between less aggressive and more aggressive tumors after liver transplantation in rats. **E** The expression of GSTA2 mRNA and protein among different HCC cell lines.

To understand the clinical significance of GSTA2 in HCC, the expression level of the GSTA2 gene was examined in the tumor and non-tumor tissues of HCC patients. The expression of GSTA2 mRNA in tumor or non-tumor tissues was significantly higher than in healthy donors (Fig. 4a). There was a significant upregulation of GSTA2 mRNA in the tumor tissues compared to the paired non-tumor issues of HCC patients (Fig. 4a). The expression of GSTA2 protein was correlated with the GSTA2 mRNA (Fig. 4b). The expression of GSTA2 mRNA in HCC tumor tissues was significantly correlated with the size of the HCC tumor and the presence of venous infiltration (Fig. 4c). The expression of GSTA2 mRNA was examined in previously established rat liver tumors under IRI treatment [6]. The expression of GSTA2 mRNA in the more aggressive rat liver tumors was significantly higher than in the less aggressive tumors (Fig. 4d). The metastatic HCC cell line MHCC97L was found to exhibit the highest expression level of the GSTA2 gene, while the hepatoblastoma cell line HepG2 was detected with the lowest levels of GSTA2 mRNA and protein (Fig. 4e).

### Overexpression of GSTA2 promoted HCC growth and aggression

**Fig. 5:**
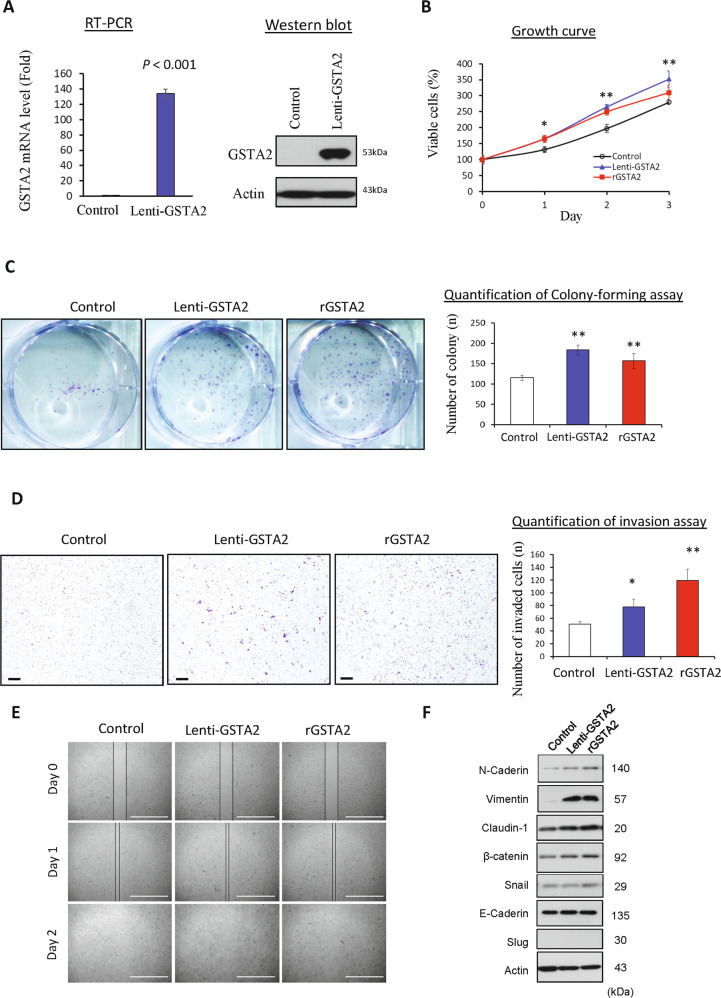
Overexpression of GSTA2 promoted HCC growth and invasion. **A** The expression level of GSTA2 mRNA and protein in GSTA2-overexpressing HCC cells. **B** MTT assay of HCC cells after endogenously overexpressing GSTA2 by lentivirus (Lent-GSTA2) or administrating human recombinant GSTA2 protein (rGSTA2). **C** Colony-forming assay. **D** Matrigel invasion assay. Scale bar: 200 μM. **E** Wound healing migration assay. Scale bar: 1000 μM. **F** Western blot analysis of EMT-associated proteins.. Control, Hep3B-control cells; Lenti-GSTA2, Hep3B-LentiGSTA2 cells; rGSTA2, human recombinant GSTA2 protein. *P < 0.05; **P < 0.01.

Established GSTA2-overexpressing stable HCC cells were validated to overexpress GSTA2 mRNA and protein compared to the control cells (Fig. 5a). The GSTA2-overexpressing HCC cells grew significantly faster than the control cells (Fig. 5b). Administration of recombinant GSTA2 protein could significantly speed up the growth rate of the HCC cells (Fig. 5b). The colony-forming ability of the HCC cells was significantly enhanced by either endogenous overexpression of the GSTA2 gene or administration of recombinant GSTA2 protein (Fig. 5c). The invasion ability of the HCC cells was enhanced when upregulation of GSTA2 either by endogenous or exogenous means (Fig. 5d), while the migration ability of the HCC cells remained similar (Fig. 5e). An increased level of GSTA2 could upregulate the expression of epithelial–mesenchymal-transition (EMT)-associated proteins including N-cadherin, vimentin, claudin, beta-catenin, and snail (Fig. 5f).

### Suppression of GSTA2 inhibited the growth and metastasis of HCC cells

**Fig. 6:**
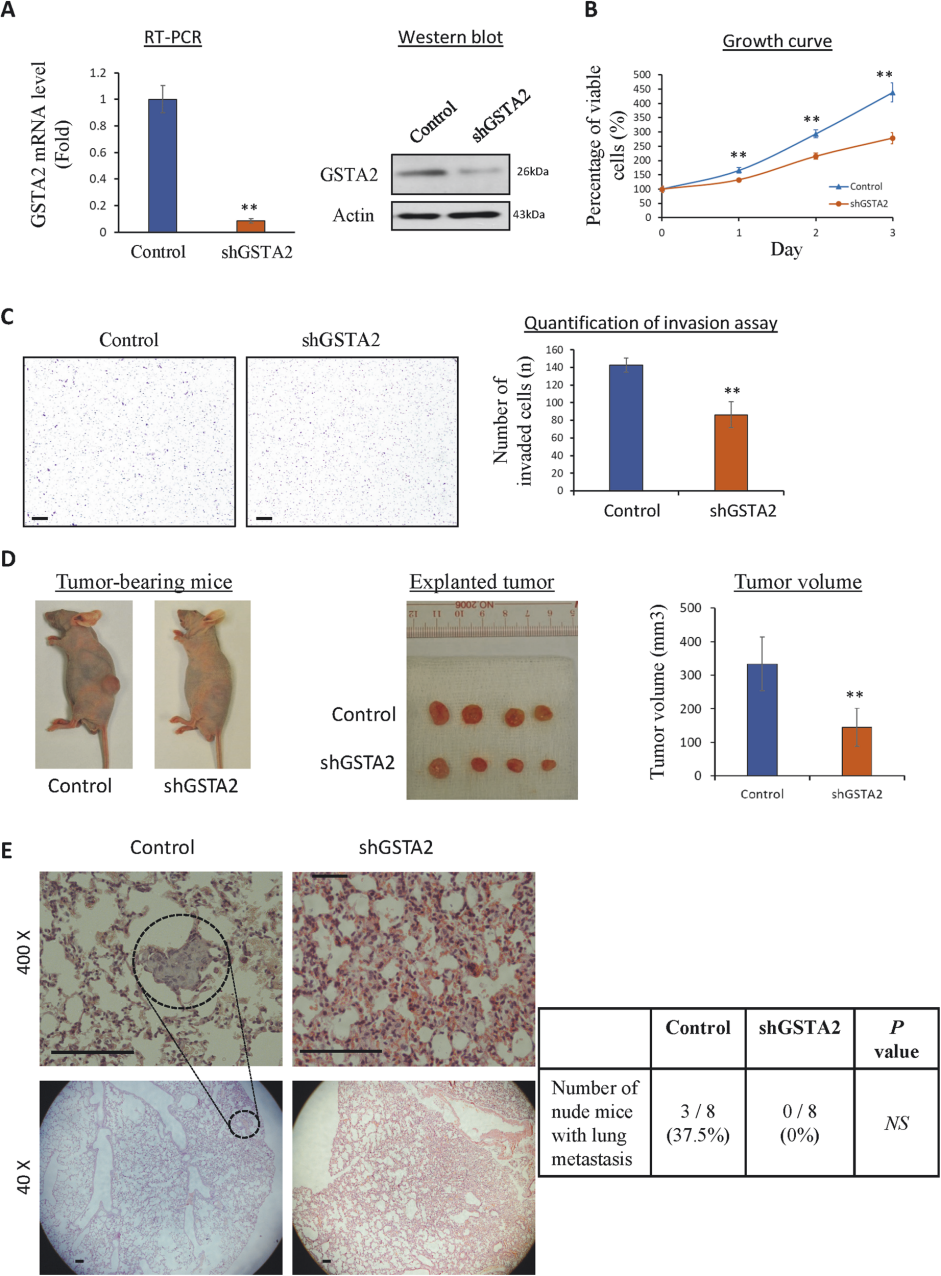
Suppression of GSTA2 expression inhibited the growth and metastasis of HCC. **A** Stable GSTA2-suppressing MHCC97L HCC cell (97L in short) exhibited significant down-regulation of GSTA2 mRNA and protein. **B** MTT proliferation assay for control and GSTA2-suppressing HCC cells. **C** Matrigel invasion assay for control and GSTA2-suppressing HCC cells. Scale bar: 200 μM. **D** Xenograft subcutaneous tumor model in nude mice. **E** H&E staining of lung tissues in metastatic tumor. The dashed circle indicates the presence of an HCC tumor in the lung. Scale bar: 100 μM. Control, MHCC97L-shControl stable cells; shGSTA2, MHCC97L-shGSTA2 stable cells; NS, no significance; *P < 0.05; **P < 0.01.

We further investigated whether targeted inhibition of GSTA2 can achieve antitumor and anti-metastasis on these malignant HCC cells. GSTA2-suppressing HCC cells were established in the metastatic HCC cells (Fig. 6a). Suppression of GSTA2 could significantly reduce the proliferation rate (Fig. 6b) and invasion ability of HCC cells (Fig. 6c). Agreed with the in vitro finding, the size of the subcutaneous in GSTA2-suppressing cells was significantly smaller than the control cells (Fig. 6d). Importantly, no metastatic tumor was detected in the lung tissue in the GSTA2-suppressing cells, while 3 out of 8 nude mice (37.5%) were found to develop metastatic tumors in the lung in the control group (Fig. 6e).

## Discussion

HCC recurrence after LT remains a significant cause of poor prognosis in HCC recipients. Expansion of donor pools in solving the problem of organ donor shortage potentially increases the risk of tumor recurrence [2, 32]. Despite employing selection strategies to minimize the HCC recipients with a potential high risk of HCC recurrence, deciphering the underlying risk factors of HCC recurrence becomes a critical task in developing effective strategies to predict and prevent HCC recurrence after LT [33, 34]. Several lines of evidence have demonstrated that the elevated systemic and regional inflammatory responses caused by IRI during the early phase increase the likelihood of late-phase tumor recurrence [4, 7, 35, 36]. Studies have also demonstrated that targeted inhibition of IRI is a conceivable strategy to diminish the risk of late-phase tumor recurrence [35, 37]. However, there are many uncovered aspects such as why and how the early phase IRI affects the risk of HCC recurrence at the later phase after LT. In this study, the GSTA2 gene was found to be the most upregulated gene in the early phase liver tissues of HCC recipients who developed recurred HCC tumors after LT. Our data showed a close relationship between the early phase circulating GSTA2 (_EPC_GSTA2) protein and hepatic as well as systemic injury at early phase after LT, indicated by its positive correlation with warm ischemic time, post-transplant levels of ROS, AST, ALT, IL-10, and IL-8, suggesting that the _EPC_GSTA2 protein may be a novel noninvasive biomarker to indicate the hepatic and systemic injury at the early phase of LT. We found that the _EPC_GSTA2 protein and USCF criteria are independent predictors of HCC recurrence after LT, indicating that both pre-transplant and post-transplant factors are critical in affecting the likelihood of HCC recurrence. Besides its potential in predicting HCC recurrence, a high level of _EPC_GSTA2 protein could significantly predict the disease-free survival of HCC recipients after LT. Altogether, our results indicated that _EPC_GSTA2 protein is a novel non-invasive biomarker not only for the indication of post-LT early phase hepatic and systemic injury but also for prediction of HCC recurrence and survival of HCC recipients.

We also found that the frequency of the nucleotide G335C SNP of GSTA2 coding sequencing, a GSTA2 protein residue S112T substitution, was significantly different between HCC-recurrence recipients and non-HCC-recurrence recipients. The percentage of heterozygous G/C alleles of G335C SNP in the recurrent group was 5-fold greater than in the non-recurrent group, indicating that this heterozygous SNP might be a potential risk factor of post-transplant HCC recurrence. Moreover, GSTA2 heterozygous G/C alleles of G335C SNP in the post-transplant liver biopsy were significantly associated with the poorest overall and disease-free survival. Therefore, we believed that assessment on GSTA2 polymorphism in the liver tissues of the donors may provide important information for stratification of high-risk recipients for closely monitoring after LT. The GSTA2 S112T serine homozygosity has been reported to be an independent factor of poor survival in acute leukemia patients who received allogeneic stem cell transplantation [38]. We postulated that the effect of GSTA2 S112T polymorphism is dependent on different clinical situations. Meanwhile, studies have demonstrated that the S112T change does not cause the change of protein structure and enzymatic activity of GSTA2 protein, but affects the level and thermostability of GSTA2 protein in the liver [39–41]. A study has also suggested that GSTA2 S112T polymorphism affects bilirubin metabolism after busulfan-conditioned allogenic transplants [38]. In our study, we did not detect a significant correlation between GSTA2 S112T polymorphism and total bilirubin in HCC recipients. We postulated that bilirubin metabolism after LT may be influenced by multifactorial factors rather than a single factor.

GSTA2 is mainly produced from the liver and functions in detoxifying electrophilic compounds such as the products of oxidative stress [16]. Our results showed that the expression of hepatic GSTA2 mRNA at the early phase after LT was significantly correlated with the expression level of ROS-regulatory genes. The level of _EPC_GSTA2 protein was also significantly correlated with the ROS level at the early phase after LT. These results thus indicated a close relationship between early phase GSTA2 and the hepatic and systemic ROS metabolism. Our results showed that elevated levels of GSTA2 protein could facilitate the normal liver cells and HCC cells to compensate their ROS level in responding to H_2_O_2_-induced oxidative stress, therefore, protected them against the H_2_O_2_-induced apoptosis and death. Moreover, overexpression of GSTA2 in HCC cells could provide a protective effect for HCC cells against high ROS-induced cell death, while suppression of GSTA2 expression in HCC cells could lead to a further elevated level of ROS and cell death in HCC cells under a high H_2_O_2_ condition. These findings agreed with another study that GSTA2 can contribute a protective effect in HCC cells against ROS cytotoxicity [16]. Our results also showed that alterations of the expression of GSTA2 could influence the ROS-associated signaling pathways such as JNK and AKT pathways in responding to the change of ROS environment. In addition, downregulation of GSTA2 expression in HCC cells led to alteration of the expression of many ROS-associated genes under the H_2_O_2_ condition. These data collectively indicated the importance of GSTA2 for HCC cells in maintaining their ROS metabolism in responding to the changes of ROS conditions. Cancer cells exhibit a phenomenon of a relatively high intracellular level of oxidative stress because of aberrant metabolism for abnormal proliferation and progression [42, 43], but a high ROS level is harmful to cancer cells, making the role of ROS in cancer remain controversial [14, 15]. Several lines of evidence have indicated that cancer cells establish a counteracting effect to high ROS by increasing the level of antioxidant enzymes to modulate the ROS level to be favorable for themselves [14, 44, 45]. Therefore, our data indicated that the upregulation of GSTA2 provides protective advantages for HCCs against high ROS-induced damage during the early phase after LT and subsequently increases the likelihood of HCC recurrence. Owing to that the ROS metabolism in cancer is complicated and regulated by the interaction among multiple cellular signaling pathways, further experiments to intensely characterize the roles of GSTA2 in multiple ROS-mediated cellular signaling pathways such as NF-κB, MAPKs, Keapl-Nrf2-ARE, and PI3-AKT, are necessary to carry out in order to understand the molecular mechanisms of GSTA2 in regulating the ROS metabolism of HCC.

The clinical relevance of GSTA2 in HCC is unknown so far. We found that the expression level of GSTA2 mRNA was commonly upregulated in HCCs and significantly correlated with tumor size and the presence of venous infiltration. The metastatic HCC cell line expressed the highest level of GSTA2 mRNA and protein over other non-metastatic HCC cell lines, implying that GSTA2 may play important role in HCC metastasis. In addition, by analyzing the HCC tissues developed from the rat LT model, we found that the expression level of GSTA2 mRNA in the more aggressive tumor tissues was significantly higher than in the less aggressive tumor tissues. Thus, the above results revealed for the first time that GSTA2 is commonly upregulated in human HCCs and positively correlated to HCC malignancy.

Functionally, we found that overexpression of endogenous GSTA2 in HCC cells could promote their proliferation rate, migration rate, and invasion ability. The administration of human recombinant GSTA2 protein could also exhibit a similar effect on HCC cells. Moreover, the increased level of either endogenous or exogenous GSTA2 protein could activate the expression of several EMT-promoting proteins including N-cadherin, vimentin, and claudin-1, while suppression of GSTA2 in the metastatic HCC cell line could inhibit its in vitro and in vivo metastatic ability. A recent study has demonstrated that targeted inhibition of glutathione and thioredoxin antioxidant pathways can synergistically kill cancer cells, suggesting that antioxidant systems are important for the initiation and progression of cancer cells [46]. Therefore, our results indicated that GSTA2 plays an essential role in metastasis of HCCs, and targeted inhibition of GST2 may be a potential therapeutic strategy for the treatment of HCC. Indeed, our animal models only demonstrated the effect of GSTA2 in HCC tumorigenesis and metastasis, further experiments by using animal LT models are essential to investigate whether targeted inhibition of GSTA2 is an effective strategy to prevent HCC recurrence after LT.

In summary, our study has demonstrated that upregulation of GSTA2 at the early phase after LT is a novel indicator of IRI and a significant risk factor of HCC recurrence. GSTA2 could be a novel prognostic and therapeutic target to combat HCC recurrence after LT.

## Materials and methods

### Patients and samples

Clinical samples were collected from 106 HCC recipients from Oct 2003 to June 2017 at the time point of 2 hr after reperfusion (early phase) in the LT operation. The last follow-up date of the recipients was in Mar 2020. There were 106 early phase plasma and 60 early phase liver biopsies were used in this study. Twenty-four liver tissues and 12 plasma samples were from healthy donors in LT. One hundred pairs of tumor and nontumor liver tissues were randomly obtained from HCC patients who received hepatectomy. All the clinical samples were obtained from the Surgical Tissue Bank of the Department of Surgery, the University of Hong Kong. A consent form was signed by each patient before sample collection. The use of clinical samples for research purposes was approved by the Institutional Review Board of the University of Hong Kong/Hospital Authority Hong Kong West Cluster (HKU/HA/HKW IRB).

### Rat liver tumor tissues

Rat liver tumor tissues generated from our previous rat liver tumor models were used [6]: (1) Less aggressive tumor group, rats were injected with tumor cells without major hepatectomy and partial hepatic I/R. (2) More aggressive tumor group: 60 min’ ischemia on the right and median lobes first, then subsequently did the major hepatectomy for left and caudate lobes (50–60% of total liver volume). After 60 min’ reperfusion (right and median lobes), tumor cells were injected via the right portal vein. Liver tumor tissues were collected from the rat after 4-week incubation.

### RNA-sequencing analysis of acute-phase liver graft in LT

RNA-sequencing analysis [22] was previously performed to compare the transcription profile of early phase liver biopsies from HCC recipients with (*N* = 6) and without (*N* = 5) post-transplantation HCC recurrence. Gene expression level between any given two samples was gauged by the number of sequencing data mapped to the gene, RPKM (reads per kilobase per million mapped reads). Each recurrence sample was compared to each non-recurrence sample to identify the upregulated and downregulated genes.

### Sanger sequencing of GSTA2 transcript

The full-length coding sequence of GSTA2 transcript (NM_000846, 669 bp, encoding 223 amino acids) was amplified by polymerase chain reaction (PCR) reaction using the FastStart Taq DNA polymerase (Roche) with the PCR condition: 95 °C, 10 min for initiate denaturation; 35 cycles of 95 °C, 1 min; 56 °C, 30 s, and 72 °C, 1 min for PCR amplification; followed by final extension step of 72 °C, 10 min. The primers used for PCR were: forward primer: 5′-CCCAAGCTCCACTACTCCAATAT-3′; reverse primer: 5′-GCAGCTTGTTCCTCAGGTTGAGTA-3′. The PCR product of GSTA2 coding sequencing of each sample was examined by gel electrophoresis. PCR products were enzymatically cleaned up by Shrimp Alkaline Phosphatase (New England Biolabs) and Exonuclease I (New England Biolabs). Samples were cycle sequenced using BigDye^™^ Terminator v3.1 Cycle Sequencing Kit (Applied Biosystems) according to the manufacturer’s protocol. Sequencing products were purified with BigDye XTerminator^™^ Kit (Applied Biosystems) and automated sequencing performed by capillary electrophoresis on an ABI 3730xl DNA Analyzer (Applied Biosystems). The Sanger sequencing analysis was conducted in the Centre for PanorOmic Sciences, LKS Faculty of Medicine, the University of Hong Kong. The Sanger sequencing data were viewed by Sequence Scanner software 1.0 (Applied Biosystems) followed by identification of single nucleotide polymorphism (SNP) by two independent researchers. The SNP genotypes were coded into three groups: homozygous (reference allele, heterozygous, homozygous (variant allele). Chi-square was used to compare the difference between recipients with and without HCC recurrence after LT. *P* < 0.05 was considered as statistical significance.

### Quantitative RT-PCR

Total RNAs in the cells or tissue samples were extracted using TRIzol^®^ reagent (Invitrogen; ThermoFisher Scientific, Inc.). RNA was converted to cDNA using a High-Capacity cDNA RT kit (Applied Biosystems; Thermo Fisher Scientific, Inc.). Quantitative PCR was performed using the Power SYBR Green PCR master mix and quantified using the ViiA7 Real-Time PCR System (Thermo Fisher Scientific, Inc.). 18S ribosomal RNA (rRNA) was used as the internal control for HCC tissue samples and β-actin was used as the internal control for cell lines. The relative expression levels of genes were determined based on our previous studies [23, 24]. Each PCR reaction was repeated twice. The primers used in this study were list in Supplementary Table S1.

### Cell lines and gene cloning

The human normal liver cell line named MIHA and the Hep3B hepatoma cell line were purchased from American Type Cell Culture (ATCC). The metastatic HCC cell line MHCC97L (97L in short) was provided by the Liver Cancer Institute & Zhongshan Hospital of Fudan University, Shanghai, People’s of Republic of China [25]. The cells lines were authenticated by STR profiling by Pangenia Lifesciences Ltd. (Kowloon, Hong Kong). The cell lines were cultured in DMEM high glucose (Gibco; Thermo Fisher Scientific, Inc.) with 10% FBS (Gibco; Thermo Fisher Scientific, Inc.), 1% penicillium and streptomycin in a 37 °C incubator supplied with 5% CO_2_.

The lentiviral open reading frame (ORF) clone of monomeric green fluorescent protein (GFP)-tagged human GSTA2 (cat. no. RC202479L2) and lentiviral ORF control clones were purchased from OriGene Technologies, Inc. Lentiviral particles were produced by transfection into HEK293 cells using Lenti-vpak Lentiviral Packaging kit (OriGene Technologies, Inc). Suppression of GSTA2 was conducted by GSTA2-specific Mission^®^ short hairpin RNA (shRNA) lentiviral transduction particles (Sigma-Aldrich; Merck KGaA). Mission TurboGFP Control transduction particles (Sigma-Aldrich; Merck KGaA) were used as a control. Lentiviral particles were transduced into HCC cells according to the manufacturer’s instructions. The expression levels of GSTA2 mRNA and protein were examined by RT-PCR) and Western blot respectively.

### ROS assay

The level of ROS, H_2_O_2_ was measured by the ROS-GloTM H_2_O_2_ Assay kit (Promega). For in vitro experiment, 5000 cells were seeded in a 96-well white plate and incubated in normal culture conditions for 24 h. The cells were treated with H_2_O_2_ for 18 h under normal culture conditions. To perform the assay, the cells were incubated with 25 µl of H_2_O_2_ substrate solution each well for 6 hours. Then 100 µl of ROS-Glo^™^ Detection Solution was applied into each well and incubated at room temperature for 20 min. The luminescent signal was quantified by a luminescence plate reader (Bio-Rad). For human serum samples, each 100 µl of serum sample was incubated with 25 µl of H_2_O_2_ substrate solution for 6 h and followed by the same step as done in in vitro experiment. Each experiment was repeated three times.

### Apoptosis assay

Apoptotic cells were stained by In Situ Cell Death Detection Kit (Roche Molecular Biochemicals, Roche Applied Science, Indianapolis, IN, USA). Cells were fixed with ice-cold acetone/methanol (1:1) at −20 °C for 10 min. The cells were digested with protease K solution (20 µg/ml) at 37 °C for 30 min. The cells were incubated with Terminal deoxynucleotidyl transferase dUTP nick end labeling (TUNEL) reaction mix at 37 °C for min. Endogenous peroxidase was blocked by 0.3% H_2_O_2_ at room temperature for 20 min. POD was applied to the cells and incubated at room temperature for 30 min. The signal was developed by DAB substrate. The apoptotic cells were captured under a microscope. An average number of apoptotic cells was calculated from five randomly selected fields. Each experiment was repeated three times.

### In vivo subcutaneous liver tumor and experimental metastasis models

The subcutaneous liver tumor model was performed as previously described [26]. HCC cells (1 × 10^6^) were suspended in 100 μl of saline and subcutaneously injected into each nude mouse (BALB/cAnN-nu, Male, 6-week old). The tumor size and body weight were measured every 5 days. After 6 weeks, the mice were sacrificed and the tumors were harvested for further analysis. Four mice were recruited for each of the experimental groups. The volume of the tumor was calculated as follows: tumor volume (cm^3^) = 1/2 × larger size × smaller size. In vivo, an experimental metastasis model was performed as previously described [26]. Cells (1 × 10^6^) were suspended in 100 μl of saline into the tail vein of nude mice. All mice were fed in standard condition with weight monitoring and sacrificed after 6-week incubation. Eight mice were recruited for each of the experimental groups. To determine lung metastases, 100 consecutive sections (4 μM) were cut and subsequently stained with H&E. The metastatic nodules were examined by an experienced pathologist and a trained researcher in a double-blinded manner. Animals were randomly selected for the experiments. The animal study was approved by the Committee on the Use of Live Animals in Teaching and Research, the University of Hong Kong (CULATR 3894-16).

### Immunohistochemistry

Each 4 μM-thick paraffin slide was dewaxed in xylene and rinsed in grade alcohol and finally rehydrated in water. Antigen was retrieved by using citric buffer (pH 6.0). The slide was blocked with peroxidase block for 20 min and 10% goat serum for 30 min respectively. Human GSTA2 antibody (LS-C166704, 1:300, LSBio) was incubated with the slide at 4 °C for overnight. A secondary antibody from the Envision system (DakoCytomation, Denmark) was applied and incubated with the slide for 30 min at room temperature. The signal was developed by a DAB substrate solution. The slide was finally counter-stained by hematoxylin solution.

### MTT viability assay

MTT viability assay was performed as described previously [27]. HCC cells (5 × 10^3^ cells per well) were seeded into a 96-well plate and incubated for 24 h. To perform MTT assay, the cells were then incubated with 100 μl of 5 mg/ml MTT solution (Invitrogen; Thermo Fisher Scientific, Inc.) for 3 h at 37 °C until crystals were formed. MTT solution was removed from each well and the crystals were dissolved in 100 μl DMSO. Color intensity was measured using a microplate reader (Bio-Rad Laboratories, Inc.) at a wavelength of 570 nm. Each experiment consisted of four replicates and at least three individual experiments were performed.

### Migration assay

The wound-healing assay was performed in the Culture-Insert 2 Well (Ibidi) culture well which contains two chambers separated by the Culture-Insert 2 Well. HCC cells (3.5 × 10e^4^ cells in 70 μl of medium) were seeded into each chamber and incubated for 24 h in normal culture conditions. The Culture-Insert 2 Well was removed to allow the formation of a gap between cells seeded in the two chambers. The cells were refilled with an appropriate volume of medium and continuously cultured for 2 days. The distance of the gap was captured under a microscope. At least three independent experiments were performed.

### Invasion assay

Invasion assay was performed by using BD BioCoat^™^ BD Matrigel^™^ Invasion Chamber (BD Biosciences). Briefly, 5 × 10^4^ cells were suspended in 500 μl of serum-free DMEM and seeded into the migration chamber. After 24 h of incubation, cells on the upper surface of the chamber were scraped out by a cotton swab. Cells migrated through the chamber were stained by hematoxylin and eosin (H&E) and subsequently counted under the microscope. At least three independent experiments were performed.

### Western blot

Total protein was extracted from cells by using cell lysis buffer (Cell Signaling Technology, Inc.). Protein lysate was quantified by using Bio-Rad Protein Assay Dye Reagent Concentrate (Bio-Rad Laboratories. Inc.). Western blotting was performed as previously described [26]. Briefly, proteins were resolved by sodium dodecyl sulfate-polyacrylamide gel electrophoresis and transferred onto polyvinylidene fluoride membrane (Millipore). Antibody at proper dilution was incubated with the membrane at 4 °C for overnight. The membrane was incubated with a secondary antibody at room temperature for 1 h. Protein intensity signal was detected using the ECL Prime Western Blotting Detection kit (GE Healthcare). Antibodies used in this study included GSTA2 (PA5-21670, 1:500, Invitrogen), β-actin (MB1501, 1: 50000, Merck KGaA), phospho-AKTser473 (#9271S, 1: 1000, Cell signaling Technologies), AKT (#9272, 1:1000, Cell signaling Technologies), and Phospho-JNK (#4671, 1:1000, Cell signaling Technologies).

### Elisa assay

The level of GSTA2 protein in plasma samples was quantified by the human GSTA2 ELISA Kit (Cat. No.: MBS941699, MyBioSources). Briefly, 100 μl of plasma sample was applied to the well of the microtiter plate followed by incubation at 37 °C for 2 h. Biotin-antibody solution (100 μl) was added to each well and incubated at 37 °C for 1 h. After the washing step, horseradish peroxidase–avidin solution was added to each well and incubated at 37 °C for 1 h. After the washing step, the TMB substrate solution was added to each well followed by incubation at 37 °C for 20 min. The Stop solution was added to each well. The optical density of each well was measured by a microplate reader and calculated as Value at 570 nm − Value at 450 nm.

### Milliplex assay

The level of cytokines/chemokines in the post-transplant early phase plasma was quantified by using the Milliplex Map Kit (EMD Millipore Corporation, MA). Analytes for detection of ten cytokines/chemokines, including GM-SCF, IFNα2, IFNγ, IL-10, IL-1β, IL-6, IL-8, IP-10, MCP-1, and TNFα were purchased from the HUMAN CYTOKINE/CHEMOKINE MAGNETIC BEAD PANEL (HCYTOMAG-60K). Each plasma sample (25 μl) and 25 μl of Assay buffer were added to each well of the 96-well micro-plate. The mixed antibody-immobilized beads (25 μl) were added to the well followed by incubation with shaking (700 rpm) at 4 °C for overnight. The plate was washed with a Washing buffer 2 times. The Detection buffer (25 μl) was added to each well and incubated at room temperature for 1 h. The Streptavidin–phycoerythrin solution (25 μl) was added to each well and incubated at room temperature for 30 min. After 2 times’ washing step, the beads were suspended with sheath fluid (150 μl) by shaking for 5 min. The median fluorescent intensity for each well was measured by the MAGPIX^®^ machine (Luminex). The concentration of each sample was calculated by a five-parameter logistic curve-fitting method.

### RT^2^ PCR array analysis

The expression level of genes critical in oxidative stress was simultaneously analyzed using an RT^2^ Profiler PCR array (Qiagen Sciences, Inc.) [28]. Total RNA extracted from HCC cells was reverse-transcribed to cDNA using the RT^2^ First Strand Kit (Qiagen Sciences, Inc.). Each Real-Time Human Oxidative Stress Plus PCR array plate (cat. no. PAHS-065YA; Qiagen Sciences, Inc.) contained 84 key genes associated with oxidative stress. qPCR was performed using the SYBR Green Master Mix (Qiagen Sciences, Inc.) on a ViiA-7 PCR machine (Thermo Fisher Scientific, Inc.). Gene expression of MHCC97L-shGSTA2 cells was calculated as the fold-difference using the 2^−ΔΔCt^ method [29] relative to the expression of MHCC97L-control cells. The mean Ct value of commonly used housekeeping genes (GAPDH and β-actin) was used as the internal control for normalization. The expression correlation between GSTA2 and the identified differential genes was analyzed by using the database of Liver HCC (369 tumor tissues of HCC patients) in the Gene Expression Profiling Interactive Analysis (GEPIA) webserver [30].

### Statistical analyses

The column-scatter plots were graphed by Prism Version 5.01 (Graphpad). The bar charts were graphed by Excel (Microsoft). Statistical analyses were performed by SPSS software version 20 (SPSS, Chicago, IL). Categorical variables were analyzed by the Chi-square test or Fisher’s exact test. Continuous variables were analyzed by Mann–Whitney *U* test or Student *t* test. The correlation analyses were analyzed by Pearson correlation analysis. To examine the prognostic value of plasma GSTA2 protein, Receiver Operating Characteristic (ROC) curve was generated for predicting tumor recurrence after LT. The optimal cut-off value of plasma GSTA2 protein was obtained from the Youden index, which was used to classify the recipients into two groups: High GSTA2 and Low GSTA2 groups. The sensitivity and specificity of plasma GSTA2 protein to predict HCC recurrence after LT was determined by ROC analysis. Logistic regression analysis was performed on significant factors in predicting post-LT HCC recurrence. Kaplan–Meier analysis applying log-rank test was performed to analyze the prognostic value of plasma GSTA2 protein in predicting overall and disease-free survival of HCC recipients after LT. Univariate Cox Proportional Hazardous Regression analysis was performed to examine the hazard ratio of plasma GSTA2 protein or clinical factors in predicting overall and disease-free survival of HCC recipients. Multivariate Cox Proportional Hazardous Regression analysis was performed to compare the significant factors in the univariate cox-regression analysis. *P* < 0.05 was considered statistically significant.

## Supplementary information

Supplementary Figure 1

Supplementary Figure 2

Supplementary Tables

Supplementary Figure Legends

## Data Availability

The correlation analysis was performed by using the database of Liver Hepatocellular carcinoma (369 tumor tissues of HCC patients) in the Gene Expression Profiling Interactive Analysis (GEPIA) webserver (http://gepia.cancer-pku.cn/detail.php?clicktag=correlation).
