# Supplementary figures and images for: Glutathione S-transferase A2 promotes hepatocellular carcinoma recurrence after liver transplantation through modulating reactive oxygen species metabolism
Source: Cell Death Discov. 2021 Jul 21;7:188. doi: 10.1038/s41420-021-00569-y (PMC8295304; doi:10.1038/s41420-021-00569-y)

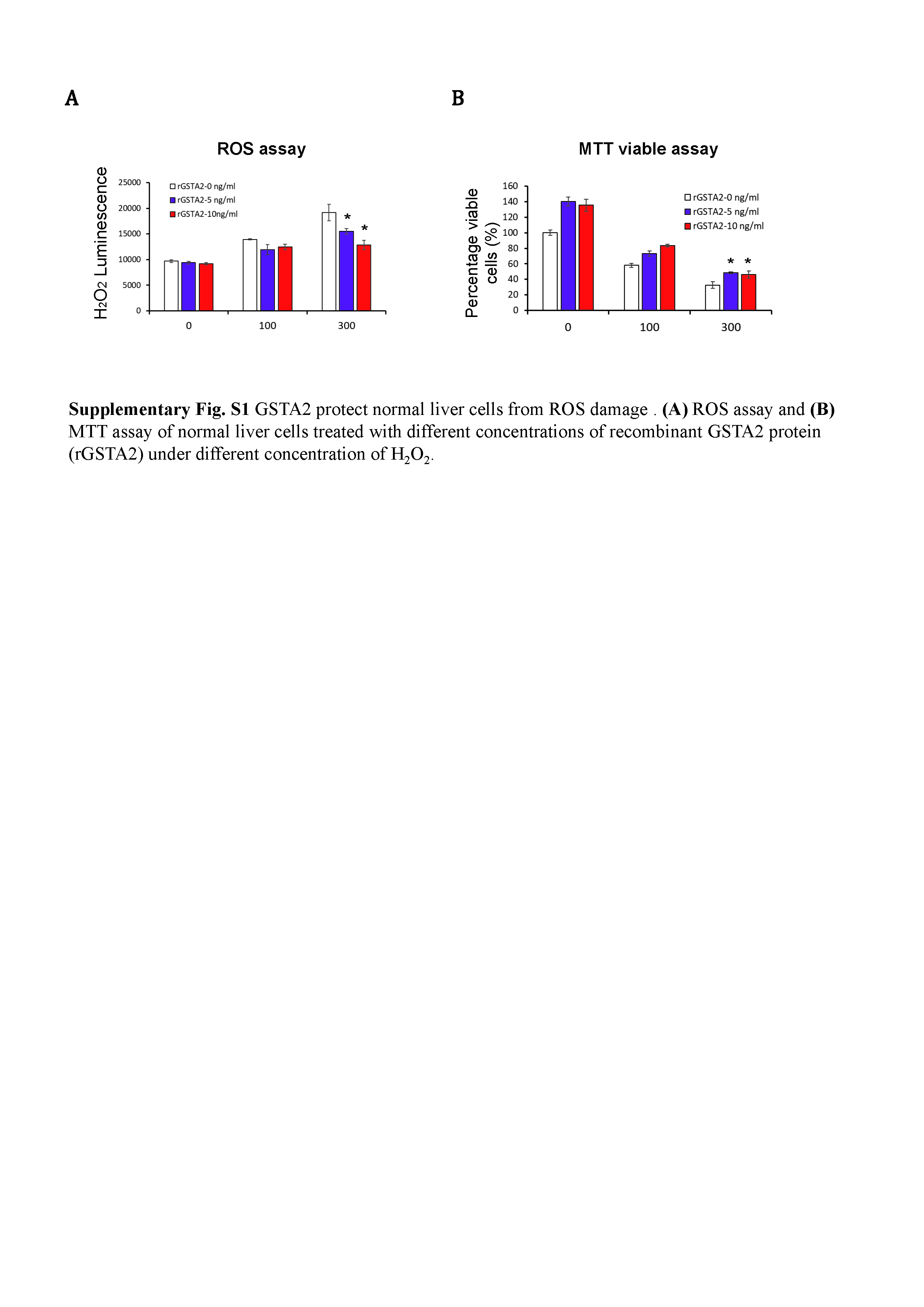

Supplement: Supplementary file 1 — Supplementary Figure 1 [file 41420_2021_569_MOESM1_ESM.tif]

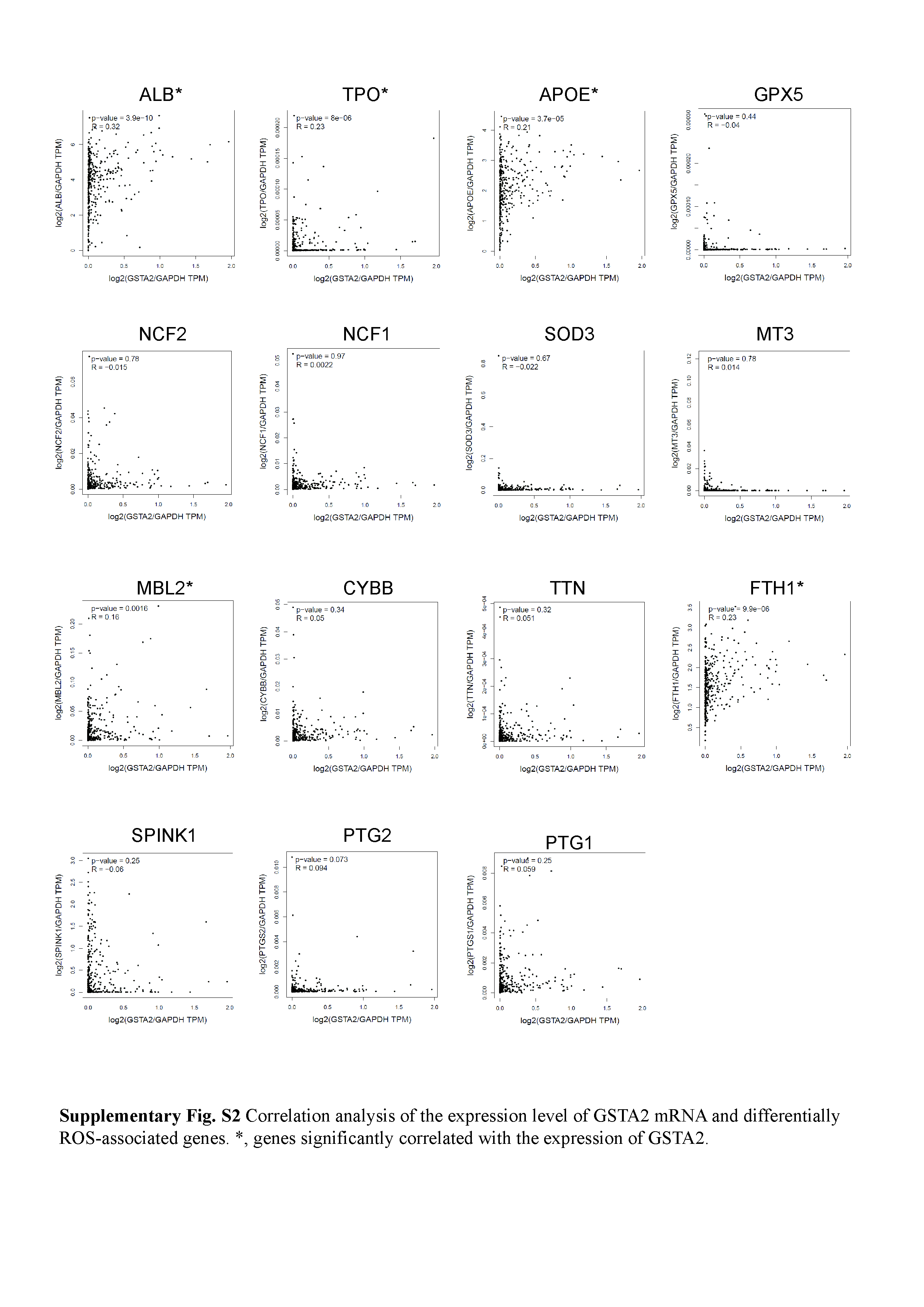

Supplement: Supplementary file 2 — Supplementary Figure 2 [file 41420_2021_569_MOESM2_ESM.tif]
