# Supplementary material for: Glutathione S-transferase A2 promotes hepatocellular carcinoma recurrence after liver transplantation through modulating reactive oxygen species metabolism
Source: Cell Death Discov. 2021 Jul 21;7:188. doi: 10.1038/s41420-021-00569-y (PMC8295304; doi:10.1038/s41420-021-00569-y)
Supplement: Supplementary file 3 — Supplementary Tables [file 41420_2021_569_MOESM3_ESM.docx]

**Supplementary Table S1** List of primers and sequences

| **Gene** | **Forward primer (5’ to 3’)** | **Reverse primer (5’ to 3’)** | **PCR size (bp)** |
| --- | --- | --- | --- |
| *GSTA2* | GACCCTTCTTTCAGTGGGAGG | CTGGAGGTTTCTCTAAGCTTTGTT | 126 |
| *TNF-alpha* | GCCCATGTTGTAGCAAACCC | GGTTATCTCTCAGCTCCACGC | 100 |
| *IL-6* | GACCCAACCACAAATGCCAG | GTGCCCATGCTACATTTGCC | 147 |
| *TGF-beta1* | GGTGGAAACCCACAACGAAAT | GAGCAACACGGGTTCAGGTA | 112 |
| *SOD3* | AGCTGGAAAGGTGCCCGA | CTTGGCGTACATGTCTCGGAT | 149 |
| *NCF1* | CCCACGGACAACCAGACAAA | CGGTACGTCTGCAGGATGAT | 104 |
| *NCF2* | GGTGCCCCTTTCAGAAGACA | GAAAGCCTTGGTCACCCACT | 101 |
| *NRF2* | ACTTCTGTTGCTCAGGTAGCC | TAAGACACTGTAACTCAGGAATGG | 103 |
| *GPx2* | AGCATGCCTTCAGGAGACAC | AACCAGAGGGTTGGGAGAGG | 118 |
| *GPx3* | GGGGATGTCAATGGAGAGAA | TTCATGGGTTCCCAGAAGAG | 113 |
| *18S* | CTCTTAGCTGAGTGTCCCGC | CTGATCGTCTTCGAACCTCC | 294 |
| *Actin* | CTCTTCCAGCCTTCCTTCCT | AGCACTGTGTTGGCGTACAG | 116 |
| *GSTA2(rat)* | TGACATGTACACCGAAGGCA | CCATGGCTCTTCAACACCTTT | 156 |
| *Actin (rat)* | AGCCATGTACGTAGCCATCC | ACCCTCATAGATGGGCACAG | 115 |

**Supplementary Table S2.** Differentially upregulated early-phase genes in HCC recipients with post-transplantation HCC recurrence

(NRc: Non-Recurrent HCC recipient; Rc: Recurrence HCC recipient)


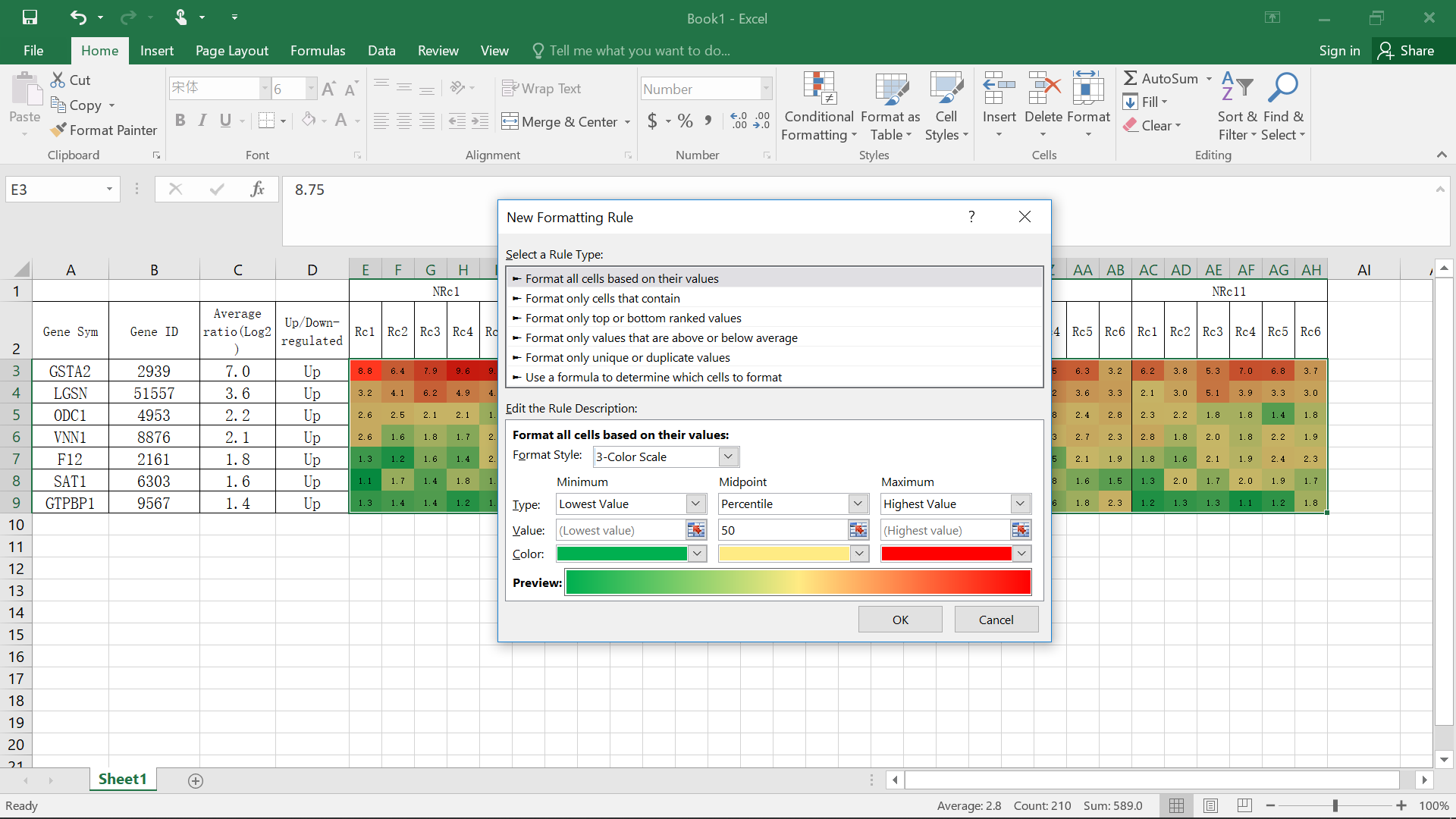


Degree of upregulation in recurrent HCC recipients

High

Low

**Supplementary Table S3** Summary of SNPs in GSTA2 transcripts between recipients with (N=6) and without (N=5) post-LT HCC recurrence by RNA-sequencing analysis

| **Chr** | **Coordinate** | **Ref** | **Alleles** | **Marker**  **type** | **HCC Recurrent**  **recipients**  **(n)** | **Non-HCC Recurrent**  **recipients**  **(n)** | **Function / amino acid**  **(mRNA sequence)** | | | |
| --- | --- | --- | --- | --- | --- | --- | --- | --- | --- | --- |
| 6 | 52,614,957 | T | C/T | Het | 2 | 0 | 3-UTR | | | |
| 6 | 52,614,978 | T | C/T | Het | 1 | 0 | 3-UTR | | | |
| 6 | 52,615,415 | T | T/G | Het | 3 | 0 | 210 | GAG > GCG | E 210 A | Non-synonymous |
| 6 | 52,617,731 | C | G/G | Hom | 1 | 2 | 112 | AGT > ACT | S 112 T | Non-synonymous |
| 6 | 52,617,731 | C | C/G | Het | 5 | 0 | 112 | AGT > ACT | S 112 T | Non-synonymous |
| 6 | 52,617,738 | G | A/G | Het | 2 | 0 | 110 | CCC > TCC | P 110 S | Non-synonymous |
| 6 | 52,617,745 | A | A/G | Het | 0 | 1 | 107 | CTT > CTC | L | synonymous |
| 6 | 52,622,717 | G | A/G | Het | 0 | 1 | 10 | TCC > TTC | S 10 F | Non-synonymous |
| 6 | 52,622,747 | G | A/A | Hom | 0 | 1 | 5-UTR | | | |
| 6 | 52,622,747 | G | G/A | Het | 1 | 0 | 5-UTR | | | |
| 6 | 52,622,755 | C | C/G | Het | 2 | 0 | 5-UTR | | | |
| 6 | 52,628,277 | C | T/C | Het | 1 | 0 | 5-UTR | | | |

**Supplementary Table S4.** Comparison of GSTA2 transcript SNPs between recipients with and without HCC recurrence after liver transplantation

| Reference allele (position in GSTA2 coding sequence) | Position of amino acid (codon change) | Codon (Amino  Acid) | Genotype | HCC Recurrent recipients  (n=13) | Non-HCC Recurrent  Recipients  (n=47) | P value |
| --- | --- | --- | --- | --- | --- | --- |
|  |  |  |  |  |  |  |
| T (28) | 10 (TCC 🡪 ACC) | TCC (Ser) | T/T (Hom) | 12 (92.3%) | 46 (97.9%) | 0.389 |
|  |  | ACC (Thr) | T/A (Het) | 1 (7.7%) | 1 (2.1%) |  |
|  |  | ACC (Thr) | A/A (Hom) | 0 (0%) | 0 (0%) |  |
|  |  |  |  |  |  |  |
| C (329) | 110 (CCC 🡪 TCC) | CCC (Pro) | C/C (Hom) | 9 (69.2%) | 38 (80.9%) | 0.086 |
|  |  | TCC (Ser) | T/C (Het) | 3 (23.1%) | 2 (4.3%) |  |
|  |  | TCC (Ser) | T/T (Hom) | 1 (7.7%) | 7 (14.9%) |  |
|  |  |  |  |  |  |  |
| G (335) | 112 (AGT 🡪ACT) | AGT (Ser) | G/G (Hom) | 0 (0%) | 12 (25.5%) | 0.002** |
|  |  | ACT (Thr) | G/C (Het) | 6 (46.2%) | 4 (8.5%) |  |
|  |  | ACT (Thr) | C/C (Hom) | 7 (53.8%) | 32 (68.1%) |  |
|  |  |  |  |  |  |  |
| A (629) | 210 (GAG 🡪 GCG) | GAG (Glu) | A/A (Hom) | 11 (84.6%) | 44 (93.6%) | 0.323 |
|  |  | GCG (Ala) | A/C (Het) | 2 (15.4%) | 2 (4.3%) |  |
|  |  | GCG (Ala) | C/C (Hom) | 0 (0%) | 1 (2.1%) |  |
