# Supplementary material for: Glutathione S-transferase A2 promotes hepatocellular carcinoma recurrence after liver transplantation through modulating reactive oxygen species metabolism
Source: Cell Death Discov. 2021 Jul 21;7:188. doi: 10.1038/s41420-021-00569-y (PMC8295304; doi:10.1038/s41420-021-00569-y)
Supplement: Supplementary file 4 — Supplementary Figure Legends [file 41420_2021_569_MOESM4_ESM.docx]

**Supplementary figure legends**

**Supplementary Fig. S1** GSTA2 protect normal liver cells from ROS damage . **(A)** ROS assay and **(B)** MTT assay of normal liver cells treated with different concentrations of recombinant GSTA2 protein (rGSTA2) under different concentration of H_2_O_2_.

**Supplementary Fig. S2.** Correlation analysis of the expression level of GSTA2 mRNA and differentially ROS-associated genes. *, genes significantly correlated with the expression of GSTA2.
